# No difference in clinical outcome after rotator cuff repair performed within or later than 3 months after trauma: a retrospective cohort study

**DOI:** 10.1007/s00167-022-07193-y

**Published:** 2022-10-26

**Authors:** Sigbjørn Dimmen, Christian Owesen, Kirsten Lundgreen, Kjersti Kaul Jenssen

**Affiliations:** 1 Orthopaedic Department, Lovisenberg Diaconal Hospital, Postboks 4970 Nydalen, 0440 Oslo, Norway; 2 University of Oslo, Oslo, Norway

**Keywords:** Traumatic rotator cuff tear, Early repair, Delayed repair, Arthroscopic rotator cuff repair, Shoulder function

## Abstract

**Purpose:**

Rotator cuff (RC) tear is one of the most common injuries of the shoulder. Patients with RC tears often report a trauma initiating shoulder pain and impaired function. The aim of this retrospective analysis of a prospectively registered cohort was to elucidate whether the time interval between the trauma and RC repair, using a cut off of 3 months, affects the functional outcome after 2 years.

**Methods:**

In a single orthopedic unit, 819 consecutive patients were treated with rotator cuff repair during the period from 2010 to 2014 and 733 of the patients completed the Western Ontario Rotator Cuff (WORC) index preoperatively and at 2-year follow-up. The Constant–Murley (CM) score was completed by trained physiotherapists after a clinical examination both preoperatively and at 2-year follow-up. Preoperative magnetic resonance imaging (MRI) was performed in all patients and postoperatively in 65% of the included patients. Re-tears and partial repairs were excluded, as were patients with pseudoparalysis who were given high priority and underwent surgery during the first 3 weeks after trauma.

**Results:**

Of the 733 treated patients, 437 (60%) reported having had a shoulder trauma in their medical history initiating their shoulder symptoms, and of these, 358 met the inclusion criteria. 296 patients with non-traumatic tears, 9 repairs done within 3 weeks after trauma, 25 partial repairs, 33 re-tears and 12 others were excluded. At 2-year follow-up there was no significant difference in WORC index (n.s.) or CM score (n.s.) between patients who had their RC repaired within or more than 3 months after trauma. In patients where RC repair was performed within 3 months, the WORC index improved by 42.9%, and in the group of patients operated later than 3 months, the increase was 38.7%. This difference between the groups was neither statistically significant (n.s.) nor clinically relevant. On postoperative MRI, 80% of the repairs were healed in both groups.

**Conclusion:**

In this retrospective cohort study, no differences in clinical outcome were found when RC repair was performed between 3 weeks and 3 months or later than 3 months after injury in patients describing their onset of symptoms as traumatic.

**Level of evidence:**

III.

## Introduction

Rotator cuff (RC) tear is one of the most common injuries of the shoulder, and patients with a RC tear report low health-related quality of life, pain and impaired function [3, 22, 27]. Whether patients should initially be treated operatively or non-operatively is debated in the literature [12, 23, 24, 31, 35, 36]. The ideal timing of RC repair after an acute trauma is also unknown. Moreover, there is no consensus on the definition of acute traumatic RC tear. In an ongoing multicenter study acute RC tear is defined as acute symptoms for less than 4 months after trauma with a full thickness supraspinatus tear documented by magnetic resonance imaging (MRI) [37]. In contrast, a systematic review concluded that the term “acute RC tear” should be used when MRI performed within 2 weeks after trauma shows muscle edema, wavelike appearance of the central part of the torn tendon and joint effusion [34]. In 1983, Bassett and Cofield reported better functional results in patients with acute traumatic RC tear who underwent open RC repair within 3 weeks compared with those repaired between 3 weeks and 3 months after injury [1]. They defined an acute RC tear as a full thickness tear after a significant injury. These findings have been confirmed in publications using both open and arthroscopic technique [17, 18].

However, if surgery is performed later than 3 weeks, it is uncertain whether the timing of the RC repair affects the clinical outcome. In a study on RC tears with pseudoparalysis, a delay of 3 months until repair had no effect on functional outcome [2]. In two similar studies, functional outcome was not affected by a surgical delay of 4 months [32, 33], while a recent retrospective study found a drop in functional outcome in patients operated more than 4 months after injury [17]. It has also been demonstrated in a study with matched cohorts that greater improvement in functional outcome occurs when repair is performed within 6 months after injury [9].

In a previous prospective study that included repairs of both chronic RC tears and tears with a history of a shoulder trauma, preoperative Western Ontario Rotator Cuff (WORC) index [21] and Constant–Murley (CM) score [7, 8] in the contralateral shoulder were demonstrated to be the best prognostic factors for increased WORC index at 2-year follow-up [19]. In that cohort study, the regression analysis did not demonstrate any difference in outcomes related to the timing of surgery, but the study did not focus specifically on patients who reported an initiating trauma.

Thus, the aim of this retrospective cohort study was to elucidate whether the timing of the repair of traumatic RC tears, using a cut off of 3 months, has any impact on patients’ functional outcome after 2 years. The WORC index 2 years after surgery was the primary endpoint, and the study hypothesis was that patients having RC surgery within 3 months would have a better clinical outcome than those repaired more than 3 months after trauma.

## Materials and methods

The study was approved by the Regional Committee for Medical and Health Research Ethics in Norway, IRB study number 0000 1870.

**Fig. 1:**
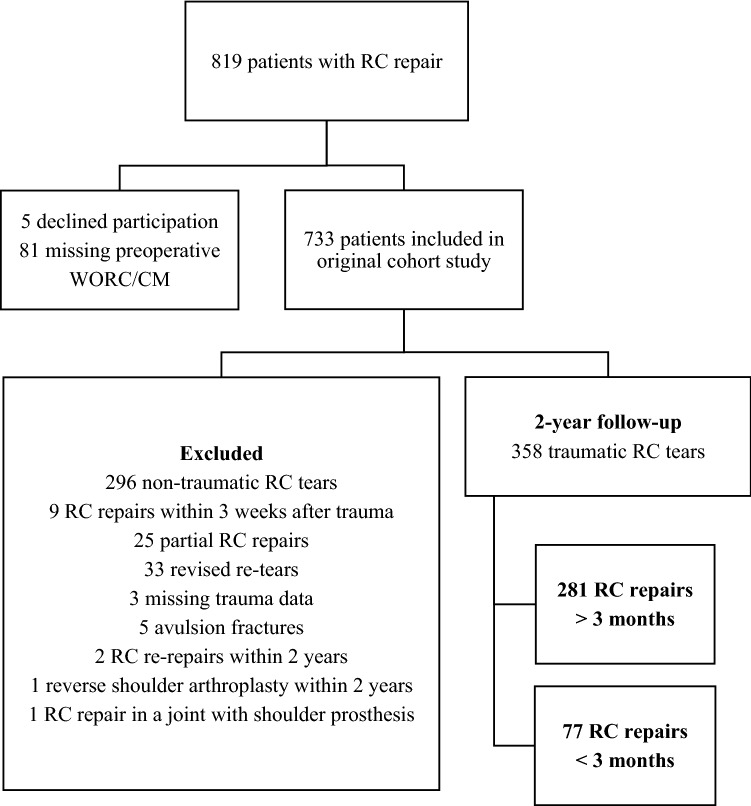
Patients flow diagram

In a prospective trial with 2-year follow-up, 733 consecutive patients treated with RC repair in our orthopedic unit during the period from 2010 to 2014 were included in the initial cohort (Fig. 1). As part of a 10-year follow-up evaluation currently being performed this secondary analysis was designed as a retrospective cohort study based on the initial prospectively registered data. All patients received written and oral information and signed an informed consent.

## Patients

Patients with RC tears, who described in their history their onset of symptoms as traumatic and who had no shoulder symptoms prior to the trauma, were divided into two groups based on the amount of time from injury to surgery; the early group had the RC repair performed within 3 months of the injury and the late group had the RC repair more than 3 months after injury. All patients referred for traumatic RC tear in this cohort were given priority for surgical repair, with a goal to do it as soon as possible and within 3 months of the trauma, or if referred later than that, within 4–8 weeks of referral. Although the traumas varied from minor distortions like lifting a heavy suitcase to high impact falls when skiing, all included patients reported a trauma as precipitating the onset of their shoulder pain and impaired function. Due to this, both patients whose tear was due to the acute trauma and patients with a previously asymptomatic degenerative rotator cuff tear that became symptomatic due to the trauma were included. Patients reporting no trauma in their medical history were considered to have a degenerative RC tear and were excluded. Also, patients having RC repair less than 3 weeks after injury due to pseudoparalysis and their need for urgent repair where excluded. Revision RC repairs and patients with partial RC repair were also excluded (Table 1).

**Table 1:** Inclusion and exclusion criteria for this analysis

Inclusion criteria	Exclusion criteria
• Enrolled in the original cohort study	• Non-traumatic RC tear
• Acute trauma with pseudoparalysis repaired within 3 weeks	• Revision of re-tear
• Completed preoperative WORC index	• Missing trauma data
• Partial RC repair	• Avulsion fracture of the RC insertion
• Preoperative CM score performed by a physiotherapist	• Re-tear and revision of already included patients within 2 years of primary repair
• Complete repair of the RC tear performed during surgery	• Revision to reversed shoulder arthroplasty within 2 years of primary repair
	• RC repair in a joint with shoulder prosthesis

Note: WORC, Western Ontario Rotator Cuff. CM, Constant–Murley. RC, rotator cuff

A total of 358 patients with traumatic RC tears were included in this analysis. In the 77 patients included in the early group, RC repair was performed 3 weeks to 3 months after their trauma (28–92 days), and in the remaining 281 patients included in the late group, repair was performed later than 3 months (93–3650 days).

## Surgical technique

In all patients the rotator cuff repairs were performed with a similar technique by specialized shoulder surgeons in our orthopedic department. With the patient in lateral decubitus position, the RC repair was done arthroscopically with 5.5-mm triple-loaded polyetheretherketone (PEEK) or titanium suture anchors (HEALICOIL PK and TWINFIX Ti; Smith & Nephew; Massachusetts; USA). A single-row technique with modified mattress suture configuration was used in all patients. Pathology of the long biceps tendon was treated with tenodesis or tenotomy. Other concomitant procedures such as acromioclavicular joint resection, subacromial decompression, coracoid resection, labral repair, capsular release and fixation of an os acromiale were performed in some patients.

The size of the tear was not measured preoperatively, but in general, one triple-loaded suture anchor was used per 15-mm tendon footprint. The number of anchors was therefore recorded as a measure of the tear size.

## Postoperative rehabilitation

After repair of small- or medium-sized tears, the patients were immobilized in a sling for 3 to 4 weeks. Patients with larger tears had a brace with a small abduction pillow for 6 weeks. Passive range of motion (ROM) was allowed from the first postoperative day, active ROM without loading was allowed after removal of the sling/brace and with loading after 3 months. Contact sports, heavy lifting and weight training were not allowed the first 6 months. Our orthopedic department’s shoulder specialized physiotherapist instructed the patients the day after surgery and at 6 weeks postoperatively. All patients received a written description of the recommended rehabilitation for their own physiotherapist who they were advised to visit 2 or 3 times a week for 3 to 6 months.

## Functional and radiological assessments

Preoperative, perioperative and 2-year follow-up data were collected. The patients completed the WORC index [21] preoperatively and at 2-year follow-up. A validated Norwegian form [10] was used. The CM score [7, 8] was determined by trained physiotherapists after a clinical examination both preoperatively and at 2-year follow-up.

Pre- and postoperative magnetic resonance imaging (MRI) was performed and evaluated by two experienced senior radiologists. Preoperative muscle atrophy was determined for each muscle according to Thomazeau classification [41]. The degree of fatty infiltration for each muscle was assessed on MRI according to the modified [11] Goutallier classification [15, 16]. The classification was done on non-fat saturated oblique T1-weighted images and the patients were divided into 2 groups for the purpose of analysis: no fatty infiltration (Goutallier grades 0 and 1) and fatty infiltration (Goutallier grades 2–4). On postoperative MRI, cuff integrity was described according to Sugaya’s classification [40]. A healed cuff was defined as Sugaya grades 1 to 3 and a non-healed cuff as Sugaya grades 4 and 5. To ensure the internal validity of the MRI data, the inter-rater agreement between the two radiologists was calculated. Gwet´s AC for inter-rater agreement on preoperative fatty infiltration was 0.66 (*p* < 0.0001), it ranged from 0.82 to 0.86 (*p* < 0.0001) for preoperative hypotrophy of subscapularis, supraspinatus, and infraspinatus and 0.91 (*p* < 0.0001) for postoperative tendon-to-bone healing. American Society of Anesthesiologists (ASA) classification and body mass index (BMI) were registered.

## Statistical analysis

Data were analyzed with SPSS software (version 25.0; IBM Corp). Group comparisons were performed for WORC index, CM score and other continuous data using independent *T*-Test. Categorical data were analyzed using cross-tabulation and compared using Pearson’s Chi-square test. *p* < 0.05 was considered statistically significant for all analyses.

The WORC index at 2-year follow-up was defined as the primary endpoint, and according to the literature, a difference between the two groups of 13 points (out of a total of 100) in the WORC index after 2 years was considered clinically relevant [10]. In this retrospective cohort study, all eligible patients in the cohort were included.

## Results

### Preoperative factors

Preoperative factors for the 358 patients included in the analysis are outlined in Table 2.

**Table 2:** Preoperative sample characteristics by timing of RC repair

Preoperative factors	Timing of RC repair after trauma		*p* values
	≤ 3 months (*n* = 77)	> 3 months (*n* = 281)	
Age at surgery, years	58 ± 10.2 (30–76)	58 ± 9.6 (17–79)	n.s
Sex			**0.002**
Women	20 (26.0%)	127 (45.2%)	
Men	57 (74.0%)	154 (54.8%)	
ASA classification	1.7 ± 0.5 (1–3)	1.9 ± 0.5 (1–3)	**0.039**
BMI, kg/m^2^	25.8 ± 3.7 (20–35)	26.9 ± 4.5 (17–43)	**0.038**
Smoke > 10 cigarettes/day	4 (5.2%)	22 (7.8%)	n.s
Previous surgery			
Same shoulder	20.8 ± 14.9 (2–79.5)	24.4 ± 15.5 (0–85)	n.s
Contralateral shoulder	75.7 ± 22.2 (5–100)	73.8 ± 19.4 (8–99)	n.s
Preoperative WORC index	42.4 ± 83.3 (1–90.1)	45.6 ± 81.1 (8.9–96.1)	n.s
Preoperative CM score	20.8 ± 14.9 (2–79.5)	24.4 ± 15.5 (0–85)	n.s
Goutallier grades 2–4	16 (20.8%)	62 (22.3%)	n.s
Thomazeau grade III	13 (17.1%)	52 (18.6%)	n.s
Number of tendons involved	1.6 ± 0.6 (1–3)	1.4 ± 0.6 (1–3)	**0.021**
1	35 (45.5%)	177 (63.0%)	
2	35 (45.5%)	86 (30.6%)	
3	7 (9.0%)	18 (6.4%)	

*ASA* American Society of Anesthesiologists, *BMI* body mass index, *RC* rotator cuff

Statistics shown are mean ± SD (range) for continuous data or *n* (%) for categorical data

A *p* value of < 0.05 indicates a significant between-group difference preoperatively

The only preoperative differences between the groups were that the late repair group had a larger proportion of women, higher ASA classification, higher BMI, and fewer tendons involved compared to the early repair group.

### Perioperative factors

Perioperative factors for the 358 patients included are outlined in Table 3.

**Table 3:** Perioperative sample characteristics and 2-year follow-up measures by timing of RC repair

	Timing of RC repair after trauma		*p *value
	≤ 3 months	> 3 months	
*Perioperative characteristics*	(*n* = 77)	(*n* = 281)	
No. of anchors used (tear size)	2.4 ± 1.1 (1–7)	1.9 ± 0.9 (1–6)	**0.001**
Biceps surgery			
None	11 (14.3%)	36 (12.8%)	n.s
Tenotomy	31 (40.3%)	151 (53.7%)	**0.036**
Tenodesis	35 (45.4%)	94 (33.5%)	n.s
Subacromial decompression	56 (72.7%)	195 (69.4%)	n.s
*2-year follow-up measures*			
*WORC index*	(*n* = 75)	(*n* = 250)	
Postoperative WORC index			
All repairs	85.3 ± 18.5 (10.7–100)	84.6 ± 18.8 (12.8–100)	n.s
1 tendon repairs	86.8 ± 19.2 (10.7–99.9)	86.1 ± 18.1 (12.8–100)	n.s
2–3 tendon repair	84.0 ± 18.0 (14.2–100)	82.0 ± 18.5 (15.0–100)	n.s
Change in WORC index (pre- to postoperative)	42.9 ± 19.5 (−10 – 89)	38.7 ± 19.7 (−10 – 86)	n.s
CM score	(*n* = 68)	(*n* = 229)	n.s
Postoperative CM score	72.7 ± 21.8 (6.3–98.3)	70.5 ± 21.5 (7.0–97.3)	n.s.
Change in CM score (pre- to postoperative)	52.3 ± 21.7 (−4.7 to 86.3)	46.6 ± 21.7 (−15.6 to 86.7)	n.s
MRI	(*n* = 57)	(*n* = 177)	
Thomazeau grade III	20 (35.1%)	56 (31.6%)	n.s
Sugaya healed	46 (80.7%)	142 (80.2%)	n.s

*CM* Constant–Murley, *MRI* magnetic resonance imaging, *RC* rotator cuff, *WORC* Western Ontario Rotator Cuff. Statistics shown are mean ± SD (range) for continuous data or *n* (%) for categorical data

A *p* value of < 0.05 indicates a significant between-group difference at the 2-year follow-up

The only perioperative differences between the groups were that the late repair group was more likely to have tenotomy performed and had smaller RC tears (based on the number of anchors used) compared to the early repair group.

### 2-year follow-up data

2-year postoperative data for the 358 patients included are outlined in Table 3. Of these, 90.8% completed the WORC index and 83.0% had a CM score at 2 -year follow-up.

**Fig. 2:**
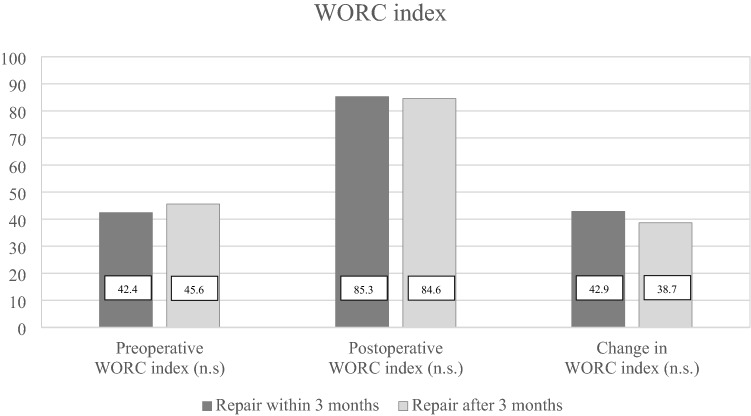
Preoperative and postoperative WORC index by timing of RC repair

**Fig. 3:**
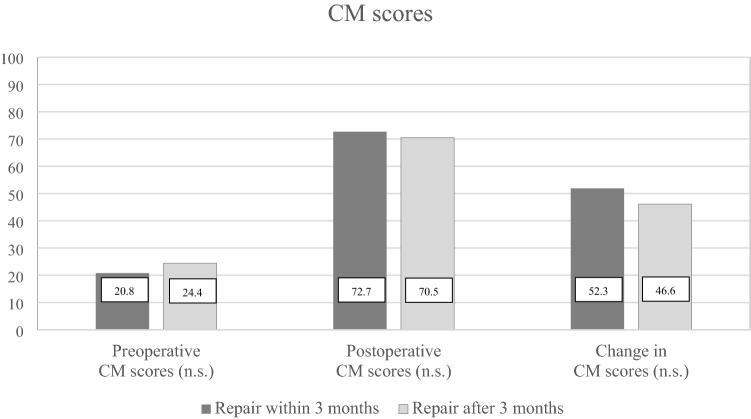
Preoperative and postoperative CM scores by timing of RC repair

At 2-year follow-up, there were no differences in WORC index (Fig. 2) or CM score (Fig. 3) in patients operated within or more than 3 months after trauma. In the early group, the WORC index increased by 42.9 (SD ± 19.5) percentage points, and in the late group the increase was 38.7 (SD ± 19.7) percentage points. In both groups, the increase in WORC index was both statistically significant and clinically relevant [10], however, the difference between the groups was neither statistically significant nor clinically relevant. Similar findings were obtained for the CM score [25].

Postoperative MRI was performed 19.7 (SD ± 8.4) months after surgery in 57 of the 77 patients (71.4%) in the early group and 21.1 (SD ± 13.7) months after surgery in 177 of the 281 patients (63.2%) in the late group. The healing rate was the same in the 2 groups, 80.7% in the group with earlier repairs and 80.2% in the group with later repairs. Furthermore, there were no difference between the 2 groups in atrophy or fatty infiltration after surgery.

## Discussion

The most important finding of the present study was that there was no difference in functional outcome, as measured by the WORC index at 2-year follow-up, in patients with traumatic RC tear repaired within or later than 3 months after trauma. Similarly, there was no group difference in CM score at 2-year follow-up.

Traumatic RC tear was defined as a full thickness tear observed on MRI in patients who reported a specific injury leading to the onset of shoulder symptoms and who had no shoulder symptoms prior to the injury. Revised re-tears, partial repairs and avulsion fractures were excluded. Nine younger patients with significant injuries and pseudoparalysis who had their RC repair performed within 3 weeks were also excluded. Consequently, the study population consisted of patients with similar RC tears, including both patients with an acute RC tear with no previous degenerative tendons, as well as those with an asymptomatic degenerative partial or full thickness RC tear before the injury, acute-on-chronic RC tear.

The choice to exclude patients operated within 3 weeks of trauma was based on the relative consensus in the literature that these patients should be treated with an acute repair [1, 17, 18].

However, after these initial 3 weeks, it is uncertain before what time point the repair should be performed. Like other authors [32, 39], we have believed that early diagnosis and repair of traumatic RC tears would improve treatment and ensure a better functional result. In our orthopedic unit, we therefore aim to perform the RC repair within 3 months after injury if the patient is referred to us in time, yet there are often both patient and physician delays that prevent this. Thus, the patient cohort was divided into 2 groups based on this cut off, those operated within 3 months of injury and those later than 3 months, to elucidate whether the functional outcome was better in those having the RC repair done early. Of the RC tears in our registry, 59.6% were classified as traumatic, a relatively high percentage compared to other reports in the literature [17, 18, 33]. There are several possible reasons for this. The registration was very accurate concerning traumatic onset of shoulder symptoms and used the criterion that the patient had no shoulder symptoms prior to the trauma. In other studies, the criteria have been much more strict, such as requiring the complete and sudden loss of shoulder function [18] or inability to achieve greater than 90 degrees of active abduction of the shoulder [33]. The 9 patients excluded from our study because they were repaired within 3 weeks of injury had this kind of pseudoparalysis.

It is generally accepted that musculotendinous retraction is the most important limitation for successful rotator cuff repair [13, 20, 30]. Shortening of the muscle fibers has been demonstrated to be the most important reason for musculotendinous retraction up to Goutallier stage 3, however, muscle fiber length remains almost unchanged from Goutallier stage 3 to 4 [29]. There is general consensus that rotator cuff repair should be done before irreversible muscular damage occurs [16]. A limitation to the present study is that retraction was not registered.

It is well known that patients with preoperative large fatty infiltration and muscle atrophy of the rotator cuff muscles have less favorable healing and functional outcome compared with patients with no fatty infiltration or muscle hypotrophy before surgery [14, 26]. Fatty infiltration and muscle atrophy have been shown to correlate with the time from injury until diagnosis of the rotator cuff tear, and greater fatty infiltration has been found in massive rotator cuff tears compared to smaller tears [28]. In a recent study of 20-year outcome after repair of massive rotator cuff tears postoperative fatty infiltration of the supraspinatus muscle was demonstrated to be a predictive parameter for postoperative CM score and tendon re-tear rate [5]. It has been demonstrated that development of Goutallier stage 2 after traumatic onset takes about 3 years on average, yet fatty infiltration can appear earlier and progress faster if more than one tendon is involved [28]. In about 20% of the RC repairs in this study, there was fatty infiltration inside the muscle, defined as Goutallier stages 2 – 4. In the original cohort which including RC repairs consecutively, distinguishing between Goutallier stages 2, 3 and 4 was not done. However, the patients included in this analysis all had stage 2 fatty infiltration, as the stage 3 and 4 fatty infiltrations were all partially repaired and therefore excluded. There was no difference in fatty infiltration in infraspinatus in patients having their repair done early or late (n.s.). According to the literature, RC repair is generally considered possible at Goutallier stage 2 [15], and it has also been demonstrated that RC repair decreases pain and improves function and strength even if re-tear is evident on MRI [20]. Due to this, our department generally attempts to repair RC tears in healthy patients with fatty infiltration stage 2.

In the two studies demonstrating no effect on outcome based on whether an RC repair was performed within 3 or 4 months after injury [2, 33], and another study demonstrating a better outcome if repair was performed within 6 months after injury [9], about 40 patients were included in each study. In a recent retrospective study including 186 patients with minimum 2-year follow-up, they concluded that RC repair should be done within 3 weeks of the injury to achieve the best results and within 4 months of injury to prevent significant functional limitations [17]. However, the differences between the groups were small. In this present study there was a larger cohort of 358 included patients, and no difference in outcome was found in RC repairs performed between 3 weeks and 3 months compared to later than 3 months after injury.

In the present study, men were significantly more likely than women to have an RC repair within 3 months of injury. This was not explained by age, as there was no age difference between the men and women. Probably, more men than women are craftsmen or similar, and this could be a reason to operate early, but the patients’ occupations were not recorded, and no conclusion can be made. Larger tears were more likely than smaller tears to be repaired before 3 months compared to after 3 months, and in this cohort men had significantly larger tears than women (*p* < 0.05). However, the same number of tendons were involved for men and women (n.s.), indicating that larger tear size among men was probably due to larger tears in infraspinatus or subscapularis. This could also explain why men were more likely to be in the early repair group, as we tend to treat RC tears involving subscapularis, and especially infraspinatus, sooner than supraspinatus tears alone to avoid development of muscle atrophy and fatty infiltration [4, 6, 14]. In a previous study, a negative correlation between postoperative muscle atrophy of infraspinatus on MRI and WORC index at 2-year follow-up was demonstrated [19]. However, in the present study, there was no significant difference in WORC at 2-year follow-up in the early versus the late group, regardless of whether only one tendon was repaired, or 2 to 3 tendons were repaired.

At baseline, patients who had RC repair more than 3 months after injury had significantly higher BMI and ASA classification than patients who underwent earlier RC repairs. However, in a previous study, we demonstrated that BMI and ASA are not correlated with functional outcome, as measured with WORC index at 2-year follow-up [19]. Based on these prior findings, the baseline differences in BMI and ASA were unlikely to have affected the results in this study.

For all patients, whether an injury led to the onset of shoulder symptoms was registered, but the severity of the injury was not. Due to this limitation, both patients with minor distortions and significant traumas were included and could not be distinguished for subgroup analysis. Another limitation is that a traumatic RC tear can involve both healthy and degenerative tendons, and it is not possible to distinguish between them unless the patient was symptomatic before the trauma. Although it was a limitation that the group sample sizes were determined by the original cohort rather than a power analysis, the relatively large number of patients is a study strength. Other study limitations include those common to all retrospective cohort studies, including the lack of data on potential cofounding factors because it was not previously collected. In the present study, there could be a problem with recall, as patients may have been incorrectly excluded if they forgot a minor trauma that may have precipitated their RC tear.

The most clinically relevant implication of the present study is that it is likely safe for most patients with an RC tear to complete 3 to 4 months of a standardized rehabilitation program before deciding to have surgery [23, 36, 38], and possibly some will achieve improved shoulder function with no need for surgical repair. Future studies with randomized controlled designs are warranted to confirm this finding. The development of muscle hypotrophy and fatty infiltration probably depends on, among other factors, whether the tendon is completely torn or there are some intact fibers in a limited full thickness tendon tear. Every patient with an RC tear should therefore be followed up closely to ensure that the muscle hypotrophy and fatty infiltration do not expand to a level that could lead to poorer surgical results.

## Conclusion

In this study there was no difference in functional outcome after RC repairs performed between 3 weeks and 3 months or later than 3 months after injury in patients describing their onset of symptoms as traumatic.
